# Revolutionizing diagnosis of pulmonary *Mycobacterium tuberculosis* based on CT: a systematic review of imaging analysis through deep learning

**DOI:** 10.3389/fmicb.2024.1510026

**Published:** 2025-01-08

**Authors:** Fei Zhang, Hui Han, Minglin Li, Tian Tian, Guilei Zhang, Zhenrong Yang, Feng Guo, Maomao Li, Yuting Wang, Jiahe Wang, Ying Liu

**Affiliations:** ^1^Department of Family Medicine, Shengjing Hospital of China Medical University, Shenyang, Liaoning, China; ^2^Science and Technology Research Center of China Customs, Beijing, China; ^3^Department of Pulmonary and Critical Care Medicine, Anshan Central Hospital, Anshan, Liaoning, China; ^4^Department of Emergency Medicine, Shengjing Hospital of China Medical University, Shenyang, Liaoning, China; ^5^Department of General Practice, First Affiliated Hospital of Anhui Medical University, Hefei, Anhui, China; ^6^Department of Cardiology, Shengjing Hospital of China Medical University, Shenyang, Liaoning, China; ^7^Department of Nephrology, Shengjing Hospital of China Medical University, Shenyang, China

**Keywords:** deep learning, pneumonia, tuberculosis, diagnosis, review

## Abstract

**Introduction:**

The mortality rate associated with *Mycobacterium tuberculosis* (MTB) has seen a significant rise in regions heavily affected by the disease over the past few decades. The traditional methods for diagnosing and differentiating tuberculosis (TB) remain thorny issues, particularly in areas with a high TB epidemic and inadequate resources. Processing numerous images can be time-consuming and tedious. Therefore, there is a need for automatic segmentation and classification technologies based on lung computed tomography (CT) scans to expedite and enhance the diagnosis of TB, enabling the rapid and secure identification of the condition. Deep learning (DL) offers a promising solution for automatically segmenting and classifying lung CT scans, expediting and enhancing TB diagnosis.

**Methods:**

This review evaluates the diagnostic accuracy of DL modalities for diagnosing pulmonary tuberculosis (PTB) after searching the PubMed and Web of Science databases using the preferred reporting items for systematic reviews and meta-analyses (PRISMA) guidelines.

**Results:**

Seven articles were found and included in the review. While DL has been widely used and achieved great success in CT-based PTB diagnosis, there are still challenges to be addressed and opportunities to be explored, including data scarcity, model generalization, interpretability, and ethical concerns. Addressing these challenges requires data augmentation, interpretable models, moral frameworks, and clinical validation.

**Conclusion:**

Further research should focus on developing robust and generalizable DL models, enhancing model interpretability, establishing ethical guidelines, and conducting clinical validation studies. DL holds great promise for transforming PTB diagnosis and improving patient outcomes.

## 1 Introduction

Tuberculosis (TB) is caused by the *Mycobacterium tuberculosis* (MTB), which predominantly targets the lungs, resulting in pulmonary tuberculosis (PTB). TB has coexisted with humans for a thousand years and approximately 1.3 million people died from TB in 2022, according to a recent report by the World Health Organization (WHO) in 2023 (Kaufmann, 2016; Ruiz-Tagle et al., 2024). There have been reports of TB across all age categories and in all nations, making it the second leading infectious killer globally, behind corona virus disease 2019 (COVID-19) (Qi et al., 2024). Furthermore, the WHO has estimated that 10.6 million individuals, including 5.8 million men, 3.5 million women, and 1.3 million children, have been diagnosed with confirmed TB infections globally (WHO, 2022). The progression of TB infection can be divided into four stages: innate immune response, immune balance, TB reactivation, and transmission. The development of effective preventative and treatment methods for this disease depends on the capacity to understand its underlying mechanisms and patterns of progression (Ernst, 2012).

**FIGURE 1:**
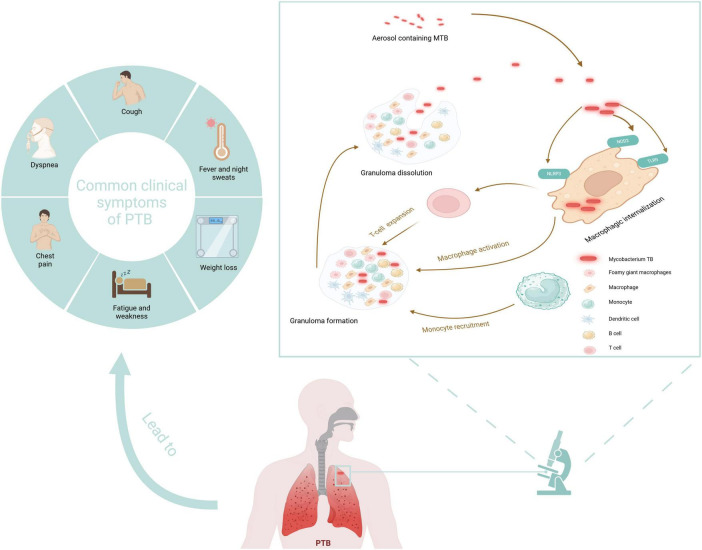
Pathogenesis and typical symptoms of TB. Created in BioRender. Zhang (2024a).

PTB involves multiple pathological processes, including inflammatory exudation, granuloma formation, necrosis absorption, fibrosis, and calcification (Xu et al., 2015). TB’s pathogenesis involves transmitting MTB by releasing aerosols containing the bacteria, which occurs when an infected individual coughs or sneezes (Figure 1). These aerosols can be inhaled by another individual, leading to infection. Upon entering the pulmonary system, MTB is phagocytosed by macrophages. Recognition of MTB components by pattern recognition receptors on macrophages, such as Toll-like receptors, initiates an immune response. Macrophages then secrete cytokines that activate T cells and promote the activation and proliferation of macrophages. The aggregation of MTB and immune cells results in granulomas forming, which confine bacterial dissemination. However, compromised immunity can lead to the breakdown of these granulomas, facilitating the recurrence and transmission of TB (Borah et al., 2021).

Artificial intelligence (AI) offers a faster and more convenient technology for predicting the effectiveness of TB treatments, especially in the detection of PTB. AI can improve the precision of medical evaluations by screening, diagnosing, and predicting outcomes with the help of imaging or clinical data (Zhan et al., 2022). A crucial part of AI is machine learning (ML), which works by training models with current data so that they can accurately predict future outcomes given past knowledge (Hussain and Junejo, 2019). Many fields of medical study have used ML, including cancer research, pharmaceutical development, illness detection, and structure of proteins prediction (Yang et al., 2022; Zhu et al., 2024). ML and deep learning (DL) have substantially contributed to computer-aided detection. Yet, DL, particularly convolutional neural networks (CNNs), has risen to prominence for detecting various pulmonary conditions, with a significant focus on diagnosing PTB. In recent years, DL, a subset of ML, has been widely adopted to develop automatic and semi-automatic systems.

DL methods are a type of hierarchical learning of representations. They are better than regular ML because they use many layers of computations to learn patterns that are not linear and have a lot of dimensions. CNNs are a type of DL design that is translation invariant. This means that once a pattern is learned, it can find it anywhere in an image, no matter where it is or how it is oriented. CNNs are often used in DL, a well-known field. These networks have many layers: input, convolutional, pooling, fully connected, and output. They can make specific predictions from digital inputs like images, sounds, genetic sequences, and clinical data (LeCun et al., 2015). Identifying PTB through imaging and ML faces several challenges, including the diverse and sometimes subtle presentations of PTB in imaging, making it difficult for ML algorithms to distinguish it from other lung conditions. ML models may overfit the training data, performing well on the data they were trained on but poorly on unseen data. As new data and knowledge about PTB become available, ML models must be updated and retrained to maintain their accuracy and relevance. Several strategies can address those challenges: employ regularization techniques to mitigate overfitting and enhance model efficacy on novel data. Utilize transfer learning methodologies in which a model initially trained on an extensive dataset is refined on a smaller, more specialized dataset to enhance performance. Extensive testing must be conducted to ensure that ML models are robust against various types of input data and can handle different imaging conditions.

This review examines seven studies about applying DL techniques in identifying PTB (Ma et al., 2020; Zhang et al., 2020, 2024; Li X. K. et al., 2021; Haq et al., 2022; Lu et al., 2022; Huang et al., 2023). The primary focus of the study is to assess the performance of various DL algorithms in diagnosing PTB. Utilize metrics such as precision, recall, F1-score, and area under the receiver operating characteristic curve (AUC) to evaluate and compare the diagnostic accuracy of different DL models. Explore strategies for integrating DL tools into clinical practice and identify the critical areas for future enhancement of the methodologies above. The outline of the review is as follows: section 2 describes the main methods of this review. Section 3 describes the traditional detection methods for PTB. Section 4 presents the DL diagnosis process for TB. Section 5 depicts the applications of DL in CT-based PTB detection. Section 6 discusses the future directions. Finally, section 7 summarizes the conclusions drawn from the review. Prior studies have not yet offered an integrated, comprehensive analysis of detecting PTB using DL alongside imaging modalities datasets. This study examines the methodologies, procedures, and techniques of DL and imaging modalities.

## 2 Materials and methods

This systematic review aimed to assess the role of DL in diagnosing PTB based on CT imaging. The preferred reporting items for systematic reviews and meta-analyses (PRISMA) guidelines were used in conducting this research (Page et al., 2021). These guidelines were instrumental in structuring the study selection, offering a standardized framework to streamline and document articles’ identification, screening, eligibility, and inclusion. A thorough search was conducted among PubMed and Web of Science databases. The search query was formulated using the PICO strategy and included the terms “tuberculosis,” “artificial intelligence,” “machine learning,” “deep learning,” “neural network,” and “natural language processing” (Cacciamani et al., 2023). In this study, the inclusion criteria were developed by the research question: in individuals undergoing diagnosis for PTB using CT scans, how effective are diagnostic models utilizing DL techniques for image analysis? This question was formulated using the PICO strategy. The population (P) comprised individuals undergoing diagnosis for PTB using CT scans, with the intervention (I) being the application of DL techniques for image analysis, and while there was no direct comparison group (C), comparisons were made with traditional diagnostic methods; the outcomes (O) focused on quantitative data regarding the performance of DL models, including primary outcomes such as accuracy, specificity, and sensitivity in diagnosing PTB. The exclusion criteria were non-English articles, letters, and reviews. Studies not related to PTB or not using CT scans for diagnosis. Studies lack a clear description of the DL methodology (studies without original data or not providing performance metrics).

**FIGURE 2:**
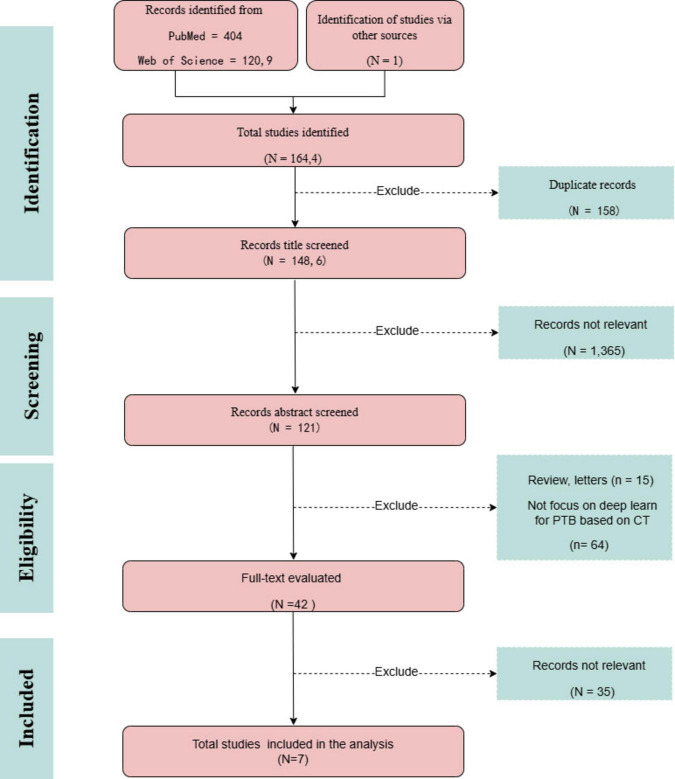
PRISMA study flow diagram.

**FIGURE 3:**
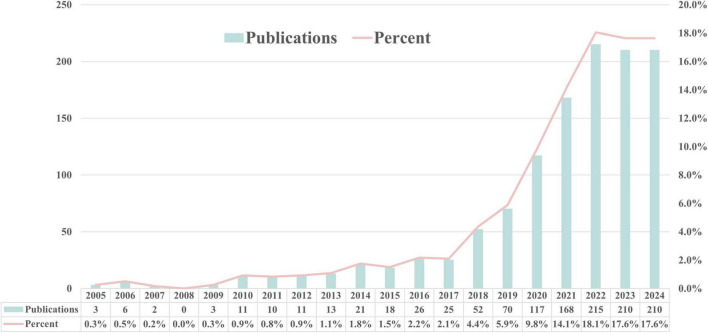
Annual number of publications of AI in TB from 2005 to 2024.

Fei Zhang and Maomao Li screened the titles and abstracts of the identified studies, and full texts were retrieved for further analysis. Any discrepancies were resolved through consensus and by consulting Jiahe Wang. Data were extracted using a predefined form to collect details on the author’s name, paper publication year, journal, country of the dataset, number of patients (with male/female distribution), study purpose, type of DL algorithm, dataset source, validation methods, reference standard, and the reported performance. The research search yielded 1,643 records; following the title and abstract screening, 120 records underwent full-text evaluation, including six publications in the review. Moreover, following citation analysis of pertinent documents, an additional article was incorporated, increasing the total number of included articles to seven. A PRISMA flow diagram was created to depict the article selection process, specifying the number of records obtained from all sources and the explanation for exclusion (Figure 2). The QUADAS-2 instrument was utilized to appraise the potential for bias within the studies under consideration (Table A1). This framework assesses bias across four principal areas (Whiting et al., 2011). Figure 3 illustrates the yearly number of publications on AI in TB from 2005 to 2024, there was a rapid increase in the publication of studies.

## 3 Traditional detection methods for PTB

### 3.1 Clinical symptoms and physical examination diagnosis

**FIGURE 4:**
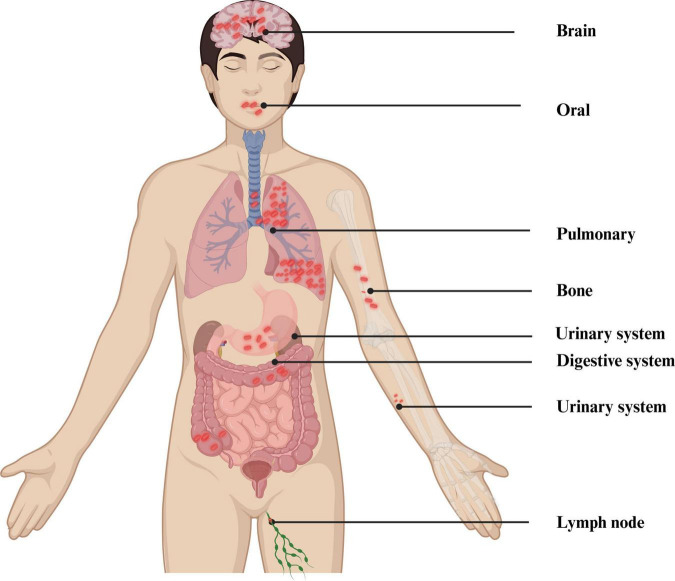
Distribution of MTB in the human body. Created in BioRender. Zhang (2024b).

Acute PTB often presents with acute respiratory symptoms, including dry cough, fever, and chest pain (Moreira et al., 2011). Symptom duration generally surpasses 2 weeks before hospitalization. The clinical appearance is comparable to non-tuberculous community-acquired pneumonia (CAP), but patients with PTB experience less intense pleural unease, toxemia, and fatigue than those with non-tuberculous bacterial pneumonia (Sharma and Mohan, 2004). Acute PTB is more likely to cause weight loss than non-tuberculous CAP, while hemoptysis is relatively rare (Morena et al., 2023). The typical symptoms of TB are vividly shown in Figure 1. Generally speaking, the diagnosis of PTB is made using a combination of conventional and contemporary techniques. This diagnosis depends primarily on the patient’s medical symptoms and findings from the physical examination, complemented by various diagnostic test results. These include bacteriological tests, the tuberculin skin test (TST), imaging studies such as X-ray or CT scans, histopathological evaluations, T-SPOT test, and the reaction to the therapy plan of antituberculosis medications (Kang et al., 2021). The distribution of mycobacterium TB in the human body is shown in Figure 4.

### 3.2 Etiologic diagnosis

The WHO regards TB culture as the “gold standard” for diagnosing TB; nonetheless, conventional solid and liquid culture media exhibit several drawbacks, such as impracticality, bacterial cross-contamination, and extended culture durations. Sputum smear microscopy is a diagnostic technique for TB that is widely acknowledged as effective (Kessel et al., 2023, pp. 2013–2017). Due to its affordability and relative simplicity compared to other advanced diagnostic methods, sputum smear microscopy remains a critical diagnostic instrument for PTB, particularly in nations with low incomes. In this process, sputum samples are coughed up by patients exhibiting symptoms and are treated with chemicals and applied to plain glass microscope slides. Yet the inconsistent clinical performance of this method, combined with the challenges in sputum collection from patients and accessibility to healthcare services, constitutes one of the main reasons for TB being undiagnosed. Subsequently, these slides are subjected to laboratory analysis to detect the presence of TB. The resulting images from a sputum smear test are typically viewed using fluorescence or light microscopy. The resolution and size of these images are determined by the level of magnification employed. For laryngeal swabs, the pooled sensitivity was 57.8% (95% CI: 50.5–65.0), and the specificity was 93.8% (88.4–96.8). For nasopharyngeal aspirates, the sensitivity was 65.2% (95% CI: 52.0–76.4), and the specificity was 97.9% (95% CI: 96.0–99.0). For oral swabs, the sensitivity was 56.7% (95% CI: 44.3–68.2), and the specificity was 91.3% (95% CI: 81.0–96.3) (Savage et al., 2023). Fluorescence microscopy offers advantages such as labor reduction and enhanced productivity. Nevertheless, the potential drawback of this technique is the danger of false-positive results, which is a result of the fluorochrome dyes’ non-specific binding (Steingart et al., 2006). The non-invasive collection process and its association with TB transmission have long been why breath has been regarded as an appealing diagnostic specimen for TB (Ghosh et al., 2021; Pham and Beauchamp, 2021). Electronic nose tests reportedly have an estimated sensitivity of 92% (95% CI: 82–97%) (Saktiawati et al., 2019).

### 3.3 Immunological diagnosis

Serological assays frequently demonstrate inadequate sensitivity and specificity, relying on a humoral immune reaction to detect antibodies against TB antigens (Steingart et al., 2011). A strategy attracting increased attention is the identification of host responses indicative of TB infection. In this regard, interferon-gamma release assays (IGRAs), such as T-Spot (Oxford Immunotec) and QuantiFERON (Qiagen), have limited use in detecting acute infections but are effective in detecting latent TB. However, IGRAs are affected by diseases such as diabetes, and the high cost also limits its application in underdeveloped regions (Takasaki et al., 2018). Currently, the T-SPOT test is extensively utilized for diagnosing infections caused by TB (Sollai et al., 2014). Despite its importance in identifying Mtb infections, a significant drawback of the T-SPOT test is that it cannot differentiate between active TB and latent TB infection (Ling et al., 2013).

The benefits and constraints of the QuantiFERON-TB Gold (QFT) test parallel those of the T-SPOT test. The QFT test offers a more straightforward operational process than T-SPOT, eliminating the need to separate peripheral blood mononuclear cells and opting to use whole blood instead (Lalvani and Pareek, 2010). Only a handful of antigenic biomarkers have been identified for TB. Lipoglycan lipoarabinomannan (LAM) is the most extensively investigated, most promising, and accessible from a simple sample such as urine (MacLean et al., 2019). The FujiLAM is a lateral flow urine test that finds LAM antigens. In adults with TB, it has a sensitivity of 70% and a specificity of 93% (Li Z. et al., 2021). The TST is a traditional diagnostic technique that uses a pure protein derivative of tuberculin to identify delayed-type hypersensitivity responses. Individuals infected with TB can generate sensitized T cells that recognize MTB antigens. Upon re-stimulation by MTB antigens, these sensitized T cells secrete different soluble lymphokines that enhance permeability, local erythema, and induration (Kowalewicz-Kulbat et al., 2018). The TST method finds the average diameter of the induration 72 h after an injection of a pure protein derivative tuberculin. An induration diameter <5 mm or no reaction is negative; ≥5 mm is positive (Abubakar et al., 2018).

### 3.4 Molecular techniques

Molecular imaging, which integrates molecular biology with medical imaging, is increasingly being explored to enhance our understanding of PTB. This high-tech imaging method allows biological processes at the molecular and cellular levels inside live things to be witnessed and measured. Techniques such as single-photon emission computed tomography (SPECT) are being investigated for their potential to offer detailed molecular-level data. SPECT’s high sensitivity and resolution make it a promising tool for identifying and tracking disease processes associated with PTB (Dimastromatteo et al., 2018). Along with smear microscopy, the Centers for Disease Control and Prevention suggests that each individual who might have PTB should also have at least one nucleic acid amplification (NAA) test, like polymerase chain reaction (PCR) (Parrish and Carroll, 2011). The high guanine and cytosine in the TB genome make it harder to conduct PCR studies. As a result, when dealing with MTB, it is essential to meticulously consider the methods for sample collection, bacterial cell disruption, nucleic acid isolation, and PCR assay design. The “Xpert MTB/RIF assay” is a rapid NAA test capable of detecting TB and determining resistance to rifampicin (Naidoo et al., 2023). The sensitivity of Xpert MTB/RIF Ultra when applied to oral swabs varied between 45% and 77.8%, in contrast to an approximate sensitivity of 90% for sputum samples (Mesman et al., 2019; Lima et al., 2020; Andama et al., 2022). Identifying bacterial RNA allows for the pinpointing of active TB. The quantification of bacterial quantity in sputum samples can be achieved using the detection of 16S ribosomal RNA, providing a sensitivity comparable to that of solid culture techniques (Honeyborne et al., 2014). Loop-mediated isothermal amplification (LAMP) is a PCR method that functions at a uniform temperature. The TB-LAMP method exhibits a sensitivity marginally lower than the Xpert MTB/RIF assay, yet both tests maintain similar specificity (Phetsuksiri et al., 2020). The WHO advocates TB-LAMP as a superior alternative to smear microscopy because of its improved diagnostic efficacy (Huang et al., 2022). A line probe test (LPA) is a quick, accurate, and flexible way to determine if someone has TB. It can detect TB in various clinical specimens, including sputum, pleural, and cerebrospinal fluid. The LPA can identify resistance to first-line TB medications such as isoniazid. Different commercial LPA kits are available, including the GenoType MTBDRplus 1.0 assay from Hain Lifescience and the INNO-LiPA Rif TB kit offered by Innogenetics (Rossau et al., 1997; Crudu et al., 2012). LPA is crucial for the management of multidrug-resistant TB. Truenat MTB, Truenat MTB Plus, and Truenat MTB-Rif Dx assays are quick molecular real-time PCR tests that can find TB. Results are usually ready in an hour (Nikam et al., 2013; Georghiou et al., 2021).

### 3.5 Imaging techniques

Most individuals with PTB exhibit abnormal chest X-ray (CXR) findings, which indicate a PTB diagnosis (Nachiappan et al., 2017). Given the relative accessibility of CXR and its utility in identifying these signs, the WHO advises using chest radiography for TB screening in populations at high risk for the disease (Liang et al., 2022). Different TB lesions appear differently on X-rays; exudative lesions appear as cloud-like or patchy shadows, proliferative lesions as nodular shadows, and caseous lesions as high-density and uneven shadows. The advantages of CT scanning include its relatively low cost, enhanced capability to differentiate between tissue types, rapid image acquisition, and broader accessibility. Compared to X-ray chest films, CT scans provide more explicit sectional images of the lungs, avoiding the issue of overlapping pictures and displaying the delicate structures and lesion details of lung tissue. Various pathological changes of TB, such as exudation, proliferation, caseation, fibrosis, and calcification, can be well displayed on CT scans. CT can detect early small lesions, bronchial dissemination foci, and mediastinal lymph node enlargement, aiding in a definitive diagnosis. It is also useful in diagnosing suspected TB cases that are atypical or negative on X-ray chest films. Additionally, CT has an extra role in assisting with fluid aspiration, biopsy confirmation, and guiding therapeutic interventional procedures, such as fluid drainage. PET/CT, which utilizes the cellular uptake of ^18^F-fluorodeoxyglucose to assess pulmonary inflammation, is highly sensitive for the early detection of TB (Ankrah et al., 2018). X-ray chests are the initial step in investigating PTB, followed by ultrasound (US), CT, and magnetic MRI for further evaluation. Additional imaging modalities, such as intravenous urography and barium studies, may also be helpful.

### 3.6 Other techniques

Innovative techniques are being developed to evaluate, track, and quantify PTB conditions at the point of care. Techniques such as lung ultrasonography and electrical impedance tomography are becoming more popular as they complement traditional diagnostic methods. These methods are being extensively researched for their potential to supplement standard procedures and, in certain respiratory conditions, to serve as an alternative due to the absence of ionizing radiation and their simplicity (Ball et al., 2017). A meta-analysis has revealed that chest US, when used for diagnosing pediatrics PTB, has a sensitivity of 84% and a specificity of 38% (Muljadi et al., 2024). Many molecular biomarkers can also be used to diagnose PTB. The combined sensitivity and specificity of monocyte to lymphocyte ratio (MLR) in detecting TB are 79.5% (95% CI: 68.5–87.3) and 80.2% (95% CI: 67.3–88.9), respectively (Adane et al., 2022).

Non-sputum biomarker tests for TB could have a market value of between $56 million and $84 million in countries with a high incidence of TB, like South Africa, Brazil, China, and India. It is thought that 14 million tests will be done (Tb Diagnostics Market Analysis Consortium, 2014, 2015; Maheshwari et al., 2016; Zhao et al., 2016). Dai et al. (2019) made a model for predicting TB by testing three iron-related biomarkers in blood serum, which can recognize TB. There is optimism that this approach could be broadened to enhance the diagnostic techniques for PTB. Next-generation sequencing is considered a revolutionary method for medication susceptibility testing of TB, providing data much faster than conventional clinical culture-based methods (Walker et al., 2015). Mass spectrometry technology can accurately detect biomarkers, which helps with the early diagnosis of TB. Chen et al. (2020) utilized Matrix-Assisted Laser Desorption/Ionization Time-of-Flight Mass Spectrometry and Liquid Chromatography-Tandem Mass Spectrometry to identify a TB-specific serum peptide signature, thereby creating diagnostic models for swift and accurate TB detection. More details can be found in Table 1, which compares various TB diagnostic methods.

**TABLE 1:** Comparison of TB methods.

Detection method	Methodology	Interpretation	Shortcomings
X-ray	A chest X-ray is taken to visualize the lungs and chest cavity.	Detects lung shadows, cavities, and calcifications.	Difficulty in distinguishing between active and latent TB, limited value for diagnosing extrapulmonary TB.
CT	A CT scan of the chest is performed to obtain detailed images of the lungs.	Detects lung inflammation, nodules, and cavities with precise localization.	Higher cost, radiation exposure, misinterpretations.
MRI	MRI scan of the chest is performed to visualize the lungs and chest cavity.	Detects lung inflammation, nodules, cavities, and soft tissue lesions.	Higher cost, less effective in detecting calcifications and bony lesions compared to X-ray.
Ultrasound	Ultrasound scan of the chest is performed to visualize the lungs and chest cavity.	Detects lung effusions, consolidations, and pleural effusions.	Requires skillful operator, lower specificity for diagnosing TB.
Bronchoscopy	A bronchoscope is used to visualize the bronchi and lung tissue, and samples are collected.	Direct visualization of lung lesions and pathological examination.	Invasive procedure with associated risks.
Tissue biopsy	Tissue samples are collected from the lungs or lymph nodes for pathological examination.	Confirms TB diagnosis and performs drug susceptibility testing.	Invasive procedure with associated risks.
Tuberculin skin test	Tuberculin is injected under the skin, and the skin reaction is observed.	Detects the immune response to *Mycobacterium tuberculosis* to determine past infection.	Cannot distinguish between active and latent TB, limited value in vaccinated populations.
Sputum smear test	Sputum is stained and examined under a microscope for the presence of *Mycobacterium tuberculosis*.	Rapid detection of active TB, low cost.	Lower sensitivity, requires multiple tests.
Fluorescent microscopy	Sputum is stained with a fluorescent dye and examined under a fluorescent microscope for the presence of *Mycobacterium tuberculosis*.	Improves the sensitivity of sputum smear test.	Higher cost, requires specialized equipment.
Culturing bacteria	Sputum or tissue samples are cultured on selective media to grow *Mycobacterium tuberculosis*.	Confirms TB diagnosis and performs drug susceptibility testing.	Higher cost, requires longer time.
Xpert MTB/RIF	Molecular diagnostic technique that detects *Mycobacterium tuberculosis* DNA in sputum and determines drug resistance.	Rapid, accurate, simultaneous detection of drug resistance.	Higher cost, requires specialized equipment.
LAMP	Rapid diagnostic technique based on nucleic acid amplification that amplifies *Mycobacterium tuberculosis* DNA within a short time.	Rapid, easy to operate.	Slightly lower specificity than Xpert MTB/RIF.
LPA	Molecular diagnostic technique based on DNA probes that detects drug resistance genes of *Mycobacterium tuberculosis*.	Rapid, accurate detection of drug resistance.	Requires specialized equipment, more complex operation.
Micro real-time PCR	Rapid diagnostic method based on real-time fluorescent quantitative PCR that amplifies and detects *Mycobacterium tuberculosis* DNA within a brief time.	Rapid, accurate, detection of drug resistance.	Requires specialized equipment, higher cost.
Next-generation sequencing	Molecular diagnostic method based on high-throughput sequencing that comprehensively analyzes the genetic information of *Mycobacterium tuberculosis*.	Detection of drug resistance and identification of new resistance genes.	Very high cost, requires specialized bioinformatics analysis.
Mass spectrometry	Molecular diagnostic method based on mass spectrometry that detects proteins or metabolites of *Mycobacterium tuberculosis*.	Detection of drug resistance, identification of new resistance mechanisms.	Very high cost, requires specialized mass spectrometry analysis.
IGRAs	Detects the level of interferon-γ in the blood to determine *Mycobacterium tuberculosis* infection.	Detection of latent TB infection.	Requires laboratory conditions, cannot distinguish between active and latent TB.
Antibody detection	Detects *Mycobacterium tuberculosis*-specific antibodies in the blood.	Assists in the diagnosis of TB.	Lower specificity, easily affected by other factors.

LAMP, loop-mediated isothermal amplification; LPA, line probe assay; IGRAs, interferon-gamma release assays.

## 4 DL diagnosis process for TB

### 4.1 Overview of DL architectures

Deep learning neural networks, a category of computational models, can learn complex feature hierarchies by deriving advanced features from simpler ones. Fukushima introduced this concept in 1980, inspired by the mechanisms of human vision based on biological principles (Yoon and Kim, 2020). DL emulates the human brain’s process of information filtering to facilitate accurate decision-making. DL instructs a computational model to handle inputs through a series of layers, analogous to the human brain’s approach, to bolster data prediction and categorization. Each layer feeds its output to the subsequent layer, akin to the progressive filtering mechanisms employed by neural networks within the brain. The iterative feedback process continues until the production remains consistent with the previous iteration. Weights are first allocated to each layer to produce the desired output, and these weights are further refined during the training process to attain the exact result (Lalmuanawma et al., 2020).

In the fields of CT-based disease diagnosis, multiple DL models are frequently utilized for tasks. CNNs like VGGNet, Google Net, ResNet, and DenseNet are essential for image categorization and feature extraction (Pavithra et al., 2023). U-Net, V-Net, SegNet, and DeepLab are commonly employed for segmentation jobs because of their proficiency in accurately delineating regions of interest (ROIs) (Yuan et al., 2022). Detection and classification jobs frequently employ models such as YOLO, SSD, and the R-CNN series, which are proficient in recognizing and categorizing objects in photos (Vilcapoma et al., 2024). GANs and their derivatives, such as DCGAN, cGAN, CycleGAN, and Pix2Pix, are utilized for data augmentation, picture reconstruction, and style transfer, improving training data’s diversity and quality (Simion et al., 2024). Three-dimensional (3D) CNNs and their variants, including 3D U-Net, are explicitly engineered for volumetric data, which is essential for examining 3D structures in CT scans (Wen et al., 2023). Furthermore, sophisticated models such as Dual-Path Networks and those developed by neural architecture search are reviewed to enhance performance in CT image processing, guaranteeing that the models are efficient and precise (Min et al., 2024).

**FIGURE 5:**
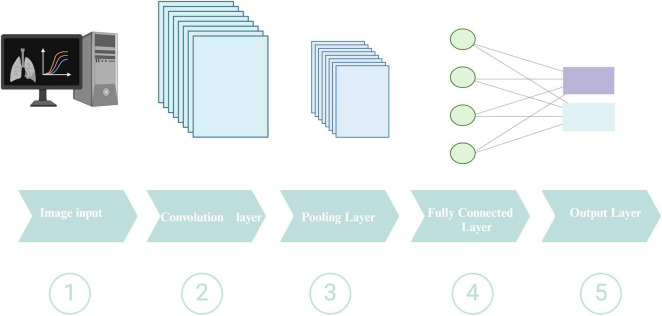
Schematic representation of a typical CNN architecture. Created in BioRender. Zhang (2024c).

Deep learning comprises three fundamental methodologies: supervised, semi-supervised, and unsupervised (Liporaci et al., 2024). For medical image analysis tasks, including disease detection and classification, CNNs have grown into the predominant DL architecture (Odimayo et al., 2024). A schematic representation of a typical CNN architecture is shown in Figure 5. Widely utilized during the diagnosis of numerous illnesses, CNNs specialize in extracting pertinent information from medical pictures, including X-rays, CT scans, and MRI scans. While CNNs excel at processing spatial information in medical images, recurrent neural networks (RNNs) and their variants, such as long short-term memory, have proven effective in handling sequential data, which is often encountered in healthcare settings (Chae et al., 2024). Generative adversarial networks (GANs) have emerged as a powerful tool to address the limited availability of labeled data by generating synthetic medical data that can be used to augment the training datasets (Su et al., 2023). Among these techniques, GANs stand out for their unique capabilities in data augmentation and image quality enhancement. GANs consist of two neural networks, a generator and a discriminator, that engage in a competitive training process. While the discriminator assesses their veracity against actual data samples, the generator generates new data instances. The generator learns to create more realistic data because of this adversarial process, which is especially useful in medical imaging where class imbalance and data scarcity are frequent problems. GANs can produce synthetic CT images of PTB that resemble actual TB lesions, increasing the data available for DL model training. This improves the model’s generalization across various patient populations and imaging settings and reduces overfitting. Semi-supervised learning methods, which leverage labeled and unlabeled data, have shown promising results in tasks like medical image segmentation and disease classification (Tseng et al., 2024). These techniques can help improve model performance when the labeled data is limited. Furthermore, unsupervised learning approaches, such as clustering and anomaly detection, have been utilized to identify novel disease subtypes, detect rare diseases, and uncover hidden patterns in medical data (Thabtah et al., 2022). These methodologies can yield essential insights for physicians and scientists, potentially facilitating the identification of novel disease biomarkers and enhanced patient screening.

### 4.2 Overview of the pipeline for PTB detection based on CT

#### 4.2.1 Data acquisition and pre-processing

**FIGURE 6:**
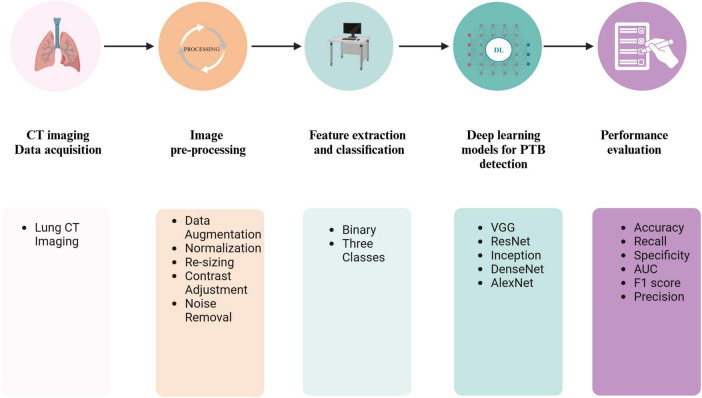
A workflow for PTB diagnosis using DL based on a CT pipeline. Created in BioRender. Zhang (2024d).

The lack of CT data might lead to data overfitting and affect the efficiency of the imaging model. Studies by Li X. K. et al. (2021) increased the number of training samples through random cropping and left-right flipping (Wu et al., 2019). A workflow for PTB diagnosis using DL based on a CT pipeline is shown in Figure 6. The initial phase of image preparation entails transforming the unprocessed images into a suitable format for subsequent analysis. Medical imaging data from various equipment exhibit dimensions, layer thickness, and scan count differences. Collectively, these variables result in a diverse array of imaging datasets, causing inconsistencies among the data sets. For accurate classification of medical images, the preprocessing phase should significantly minimize noise without compromising the integrity of vital image elements. Consequently, the preprocessing phase consists of resizing, normalizing, and occasionally converting color images from RGB to grayscale. Additionally, images are enhanced using techniques such as Gaussian blurring, median filtering, morphological smoothing, and various other methods for image adjustment.

#### 4.2.2 Feature extraction and classification

The process of transforming images into features that reflect various image attributes is called feature extraction. The success of DL models in CT-based diagnosis is heavily dependent on the quality of annotated data used for training and validation. In this systematic review, CT scans were annotated by experienced radiologists or pulmonary disease specialist following standardized clinical guidelines (Ma et al., 2020; Zhang et al., 2020, 2024; Li X. K. et al., 2021; Haq et al., 2022; Lu et al., 2022; Huang et al., 2023). Image segmentation is indeed a pivotal step in image processing. It involves partitioning images into distinct sections or ROIs to isolate and analyze specific features or objects within the image (Shahzad et al., 2024). Several types of features are used in image analysis. These include texture, shape, contrast, and brightness (Kaifi, 2023). Slices exhibiting PTB lesions and regular slices devoid of pathological findings were individually marked manually and employed as the benchmark dataset to train the DL model. Some open-source tools, such as ITK-SNAP, delineate bounding boxes around CT imaging lesions (Yan et al., 2021b). This process entails identifying and segmenting the lesions from the scans. Nevertheless, manual segmentation of the lung region is a laborious, monotonous, and time-consuming endeavor that significantly depends on the proficiency and experience of radiologists. Feature extraction is a cornerstone of diagnostic imaging, particularly when utilizing DL to analyze CT scans.

Gordaliza et al.’s (2019) study offers a glimpse into the potential of unsupervised learning for lung image segmentation. According to Wang et al. (2023), they created a GAN-based design that can separate different lung lesions. GAN is an ML model made up of a generator and a discriminator. It is often used to create images and split them into groups. This model can identify and segment multiple lesion areas present in CT scans. In a GAN, the discriminator may experience “forgetting,” which means losing the ability to recognize certain features during training. They implement a method to mitigate this forgetting phenomenon. It introduces a self-supervised rotation loss to help address the issue of discriminator forgetting. Self-supervised learning is free of data to be labeled by hand, and rotation loss might include flipping pictures to aid the model in learning better. The recommended approach achieved Dice coefficients of 68.5% on test datasets for multi-center PTB. The architecture consists of a dual attention module and a cascaded context-aware pyramid feature extraction method, making it possible to understand the semantic dependencies linked to lung lesion characteristics in space and time. This unified method makes the model’s training more effective. The study by Gordaliza et al. (2019) develops a methodology for the automated extraction of a radiological biomarker from CT scans to assess the disease burden of TB, which may also be modified for pneumonia identification. The pipeline involves lung segmentation, tissue type classification, and applying a Gaussian mixture model (GMM) to differentiate between healthy and diseased tissue. The process consists of using an adaptive thresholding method to identify air-like organs in chest CT scans, such as healthy lungs, the airway tree, and the stomach, by utilizing the topological properties of the organs. Geodesic Active Contours are pivotal in refining the lung boundaries by including lesions attached to the pleura and discarding motion artifacts. Furthermore, the GMM is employed to model the probability distribution of voxel intensities within the segmented images. By assuming that the tissue intensities follow a Gaussian mixture, the GMM, in conjunction with the Expectation-Maximization algorithm, allows for the automatic computation of thresholds that distinguish different tissue types. This statistical approach provides a robust framework for classifying lung tissue based on its intensity values.

#### 4.2.3 Performance evaluation

The effectiveness of the entire pipeline is measured using evaluation metrics such as precision, accuracy, recall, specificity, F1-score, and AUC, among others. The training subset is utilized to generate a specific model. In contrast, the suitability of the training process and the model is evaluated by simultaneously observing overfitting or underfitting on the validation subset. Ultimately, the unseen testing subset is used to judge the performance to which the created model works. Sensitivity is the ratio of accurate positive results to the actual positive cases. Specificity refers to the proportion of true negative cases that are accurately recognized as such. The Jaccard index (JI) is a percentage that shows how much the model’s predicted output and the accurate annotation ground-truth mask match. The similarity index measures the unity between the segmentation generated by the model and the expert-annotated ground truth. It evaluates the extent to which the model’s delineation of the PTB region aligns with the input image’s actual PTB area. A Dice similarity coefficient (DSC) of zero indicates no spatial overlap between the model’s annotations and the actual PTB location, while a DSC of one signifies perfect spatial overlap. The AUC summarizes the receiver operating characteristic (ROC) curve. The ROC curve compares the sensitivity to the false positive rate to see how well a classifier can tell the difference between classes. Additional details can be found in Table 2, which provides some standard performance metrics for DL models.

**TABLE 2:** Common performance metrics for DL models.

Metric	Description	Formula
Accuracy	The ratio of correctly predicted instances to the total instances	Accuracy = (TP + TN) / (TP + TN + FP + FN)
Precision	The ratio of true positive predictions to the total positive predictions	Precision = TP / (TP + FP)
Recall	The ratio of true positive predictions to the total actual positives	Recall = TP / (TP + FN)
Specificity	The ratio of true negative predictions to the total actual negatives	Specificity = TN / (TN + FP)
Kappa	A statistic that measures inter-rater agreement for categorical items	Kappa = (P_o_ − P_e_) / (1 − P_e_)^1^
F1-score	The harmonic mean of precision and recall, balancing both metrics	F1-score = 2 × precision × recall / (precision + recall)
Dice similarity coefficient	A measure of overlap between two sets, often used in image segmentation tasks	Dice = (2 × TP) / (2 × TP + FP + FN)

^1^P_o_ is observed agreement, and P_e_ is expected agreement.

#### 4.2.4 DL-related concepts

Deep learning is a subset of ML that focuses on deep artificial neural networks (ANNs). Common types of DL algorithms encompass multi-layer perceptrons (MLPs), CNNs, RNNs, graph neural networks, Transformers, and more. Overfitting is a modeling error that arises when a model learns the random noise and fluctuations in the training data to the extent that it negatively impacts the model’s performance on new, unseen data. Essentially, the model becomes too tailored to the training set and fails to generalize well to independent data sets, such as those used for testing. Cross-validation is a way to see if the outcomes of a statistical test can be applied to a different data set. It is mainly used when the goal is to make a prediction and figure out how well a prediction model will work in real life. In *k*-fold cross-validation, the original sample is split into *k* subsamples of the same size. Only one *k* subsamples are kept as confirmation data to test the model, and the other *k* − 1 subsamples are used as training data. After that, this process is done *k* times, and each *k* subsample is used only once as confirmation data. In leave-one-out cross-validation, one observation from the sample is used as the validation set, and the rest are used as the training set. This is a type of *k*-fold cross-validation, where *k* is the total number of data points. Because this is done repeatedly, each measurement in the dataset is used as the validation set a single time. The same dataset is used for training and validation in cross-validation, so it is an internal validation method. External validation, on the other hand, uses a different set of data that was not used to train and test the model in the first place. This could involve data from a different time, location, or group of subjects. In the bootstrap validation technique, for every iteration, a subset of the original dataset is selected randomly with replacement to serve as the training dataset for the model. The data points not included in the training subset, known as the out-of-sample points, constitute the validation set. This procedure is conducted n times consecutively, and the average error rate from these n iterations is calculated to assess the model’s predictive accuracy.

## 5 Applications of DL in CT-based PTB detection

Multiple DL algorithms have been widely applied to CT-based PTB diagnosis, including 3D CNN, MLP, U-Net, DTE-SVM, ICNN, and GNN. These algorithms have unique feature extraction and classification capability characteristics suitable for different datasets and diagnostic tasks. U-Net is often used for medical image segmentation tasks and performs well in segmenting and diagnosing PTB lesions. Ma et al. (2020) utilized U-Net to process CT data from 337 ATB cases, 110 pneumonia cases, and 120 healthy individuals. Utilizing an independent dataset for testing, they achieved exceptional results, demonstrating a positive predictive value of 0.971 and an AUC score of 0.980.

Three-dimensional CNNs can process 3D chest CT data, fully exploiting spatial information for diagnosis. Li X. K. et al. (2021) developed a 3D CNN to assist in diagnosing PTB, achieving 93.7% precision and 98.7% recall by learning from 501 PTB patients and an equal number of standard samples. Most studies employ cross-validation methods like 5-fold or 10-fold cross-validation. For example, Zhang et al. (2024) used MAResNet with 905 chest CT samples provided by Beijing Chest Hospital, adopting fivefold cross-validation, achieving an accuracy of 94% with sensitivity and specificity reaching 93.80% and 94.20%, respectively. Haq et al. (2022) used an ANN-based classifier, MLP, with 10-fold cross-validation, achieving an accuracy of 99% and a very high Kappa coefficient (0.98). Some studies use independent test data for model performance evaluation, such as in the study by Li X. K. et al. (2021). Open data sharing can accelerate the development and validation of CT-based PTB algorithms, improving research validation capabilities. The study by Huang et al. (2023) illustrates this value well; their research is based on the data released by Zhang et al. (2020). They employed a DTE-SVM algorithm, showing satisfactory results in terms of accuracy and sensitivity. In conclusion, these research findings demonstrate that as DL algorithms mature, their application in CT-based PTB diagnosis is becoming increasingly widespread and practical. Characteristics of the included studies are shown in Table 3.

**TABLE 3:** Characteristics of the included studies.

References	Journal	Country	Number of patients (male/female)	Purpose	Deep learn algorithm type	Dataset source	Dataset	Validation	Reference standard	Performances
Zhang et al., 2024	Medical & Biological Engineering & Computing	China	N/A	Diagnosis of PTB with 3D neural network	MAResNet	Beijing Chest Hospital	905 chest CT scans (500 PTB vs. 405 normal)	Fivefold cross-validation	Radiologist annotation	Accuracy: 94%; sensitivity: 93.80%; specificity: 94.20%; AUC: 0.97
Haq et al., 2022	Symmetry	Pakistan	N/A	Diagnosis of PTB	ANN based classifier MLP	Bahawal Victoria Hospital	200 chest CT scans (100 PTB vs. 100 normal)	10-fold cross-validation	Pulmonary disease specialist label	Accuracy of 99%; kappa: 0.98
Ma et al., 2020	Journal of X-Ray Science and Technology	China	518/328	Diagnosis of ATB	U-Net	Hebei University Affiliated Hospital	337 ATB, 110 pneumonia, and 120 normal cases	Independent test data containing 139 ATB, 40 pneumonia, and 100 normal cases	Sputum smear for ATB patients; CT report result for normal and pneumonia patients.	Accuracy: 0.968; sensitivity: 0.964; specificity: 0.971; positive predictive value: 0.971; negative predictive value: 0.964; AUC: 0.980
Huang et al., 2023	IEEE/ACM Transactions on Computational Biology and Bioinformatics	N/A	88/46	Diagnosis of PTB	DTE-SVM	Hospital database	288 CT images (144 PTB, 144 normal)/68 PTB and 66 normal	10-fold cross-validation	Radiologist	Accuracy: 94.62% ± 1.00; F1-score: 94.62% ± 1.00; precision: 95.30% ± 1.24; sensitivity: 93.89% ± 1.96; specificity: 95.35% ± 1.31; AUC: 0.9579
Zhang et al., 2020	Journal of Ambient Intelligence and Humanized Computing	N/A	88/46	Diagnosis of secondary PTB	ICNN	Hospital database	144 CT imaging datasets from 68 secondary PTB and 144 CT image datasets from 66 normal people	Independent test data containing 29 secondary PTB and 29 normal images	Radiologists	Accuracy: 93.95%; sensitivity: 94.19%; specificity: 93.72%
Li X. K. et al., 2021	Applied Intelligence	China	N/A	Diagnosis of PTB	3D CNN	Affiliated Hospital of Zhejiang University	501 CT imaging datasets from 223 PTB and 501 CT image datasets from normal people	Five-folder cross-validation and independent test data (containing 75 PTB and 75 normal cases)	Radiologist label	Precision = 93.7%, recall = 98.7%
Lu et al., 2022	Computer Methods and Programs in Biomedicine	China	N/A	Diagnosis of PTB	Graph neural network	Fourth Hospital of Huai’an	840 chest CT scans (420 PTB vs. 420 normal)	Fivefold cross-validation	Radiologist	Accuracy: 98.93%; sensitivity: 100%; specificity: 97.94%; precision: 97.86%; F1 score: 98.91%

PTB, pulmonary tuberculosis; HC, healthy controls; MAResNet, multi-scale attention ResNet; ANN, artificial neural network; MLP, multi-layer perceptron; ATB, active tuberculosis; DTE-SVM, deep transferred efficientNet with SVM; CNN, convolutional neural network; ICNN, improved convolutional neural network.

### 5.1 Detection and classification of TB lesions

**FIGURE 7:**
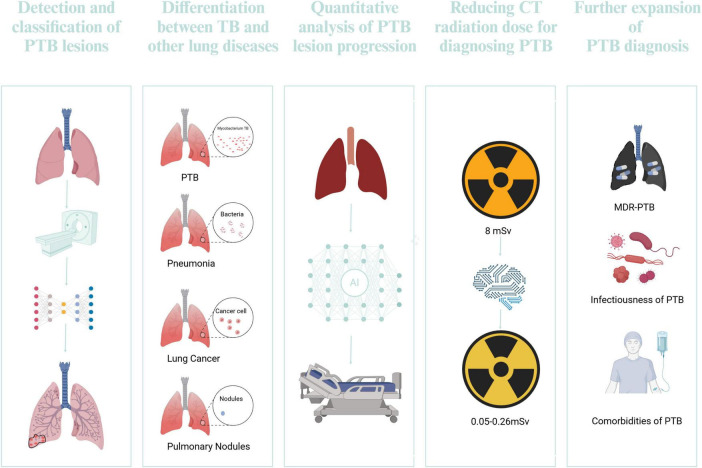
The application of DL in TB management. Created in BioRender. Zhang (2024e)

Conventional techniques for PTB detection frequently depend on radiologists’ expertise, which may be subjective and protracted. The integration of DL into this process has shown promising advancements. The application of DL in TB management is shown in Figure 7. Zhang et al. (2024) introduces a 3D multi-scale attention residual network (MAResNet) to recognize PTB utilizing CT images. MAResNet is the integration of the Convolutional Block Attention Module (CBAM) alongside residual modules. This dual mechanism enhances the distinguishability of image features and allows for the efficient reuse of shallow features. The accuracy of MAResNet in classifying PTB reaches 94%, which is essential for differential diagnosis and treatment planning.

Another study by Yoon et al. (2023) a 3D neural network model, nnU-Net, will be created to investigate the clinical significance of CT cavity volume and evaluate the model’s efficacy in cavity detection. The research retrospectively analyzed 392 patients with mycobacterial pulmonary disease, including TB and non-tuberculous pulmonary disease. The nnU-Net model demonstrated high sensitivity in detecting cavities, with a mean DSC of 78.9. One notable application is the cascading deep supervision U-Net model, as highlighted in the study by Hu et al. (2022), which concentrates on the diagnosis of pneumoconiosis complicated by PTB. The CSNet model leverages the strengths of HRCT to provide high-resolution imaging coupled with the DL framework to enhance the segmentation and diagnosis of affected lung tissues. This approach has shown superior performance over traditional U-Net models, with an AUC value of 0.947.

### 5.2 Differentiation between TB and other lung diseases

#### 5.2.1 Distinguishing PTB and non-tuberculous mycobacteria lung disease

Wang et al. (2021) retrospectively amassed chest CT images from 301 patients with non-tuberculous mycobacterial lung disease (NTM-LD) and 804 patients with PTB. The definitive diagnostic criterion was pathogenic microbiological analysis. They utilized a 3D ResNet model, attaining AUC scores of 0.90, 0.88, and 0.86 for the training, validation, and testing datasets. Additionally, when assessed on an external dataset consisting of 40 cases of NTM-LD and 40 cases of MTB-LD, the AUC was 0.78. The 3D-ResNet model had a markedly enhanced capacity to distinguish between the two circumstances relative to radiologists with 10 years of expertise, and its diagnosis speed surpassed that of the radiologists by more than 1,000 times.

#### 5.2.2 Distinguishing PTB and pneumonia

The advent of DL has revolutionized the field of medical imaging, particularly in the differentiation between PTB and CAP. Han et al. (2023) have used the power of 3D-CNNs to discern PTB from CAP using chest CT images. Their model was trained and validated using a dataset comprising 493 patients from two imaging centers. The model achieved an accuracy of 0.989 in the internal and 0.934 in the external test set, showcasing its robustness in differentiating the two conditions. The ability of the 3D-CNN to directly extract abstract features from images without the need for manual segmentation aligns with the growing trend in radiomics, which relies on high-throughput feature analysis. This method accelerates the diagnosis process and reduces the impact of subjective interpretation prevalent in conventional radiological evaluations.

#### 5.2.3 Distinguishing PTB and lung cancer

Lung cancer can be categorized into three main groups. Distinguishing PTB from lung cancer is challenging due to their overlapping clinical and radiological features. Feng et al. (2020) used a CNN method to extract features from CT images, creating a DL signature to predict the likelihood of PTB or lung adenocarcinoma. They also developed a DL nomogram combining the DL signature with clinical factors and CT-based findings. The DL nomogram showed impressive AUCs of 0.889 in the training set, 0.879 in the internal validation set, and 0.809 in the external validation set. Tan et al. (2022) utilized a customized VGG16 model trained with transfer learning and achieved an accuracy of 90.4% in distinguishing between TB lung nodules and lung cancer. The accurate differentiation between TB and pulmonary nodules in CT images is crucial for effective diagnosis and treatment planning. Conventional techniques frequently depend on the proficiency of radiologists, which may be subjective and labor-intensive. The advent of DL has introduced a paradigm shift in this domain, offering automated and efficient solutions.

### 5.3 Quantitative analysis of PTB lesion progression

In addition to identifying PTB, DP is adept at monitoring changes in patients’ conditions following treatment and assessing the severity of the disease.

The accurate quantification of lesion progression in PTB from CT images is pivotal for disease monitoring and treatment response evaluation. The integration of depth information in the ResNet model allowed for capturing the 3D characteristics of pulmonary lesions, providing a more comprehensive analysis than traditional 2D image assessments. This approach underscores the potential of DL in identifying the presence of disease and quantifying its extent and severity. Gao et al. (2020) developed a 3D ResNet incorporating depth information at each layer, the suggested depth-ResNet model demonstrated remarkable performance, with an average classification accuracy of 92.7% in predicting severity scores. Wu et al. (2019) utilize DL to create a diagnostic framework that detects PTB lesions and classifies them into specific types, such as military and tuberculoma. Applying a Noisy-Or Bayesian function to calculate an overall infection probability enhances the diagnostic report with quantitative analysis, offering clinicians a more thorough comprehension of the infection’s scope and characteristics. The approach utilized advanced 3D CNNs to examine CT imaging datasets from 233 patients with active PTB and 501 healthy controls. The recall and precision for identifying PTB patients were 98.7% and 93.7%, respectively. The classification accuracy of PTB was 90.9%.

### 5.4 Reducing CT radiation dose for diagnosing PTB

Studies have shown that the effective radiation dose from a single CT scan can range from a few millisieverts (mSv) to over 8 mSv, which is significantly higher than the typical annual background radiation exposure of around 3 mSv (Sharma and Surani, 2020; McKenna and McMonagle, 2024). Using ionizing radiation in CT scans has raised concerns about the potential health risks. The mean radiation exposure for ultra-low-dose computed tomography (ULDCT) ranges from 0.05 to 0.26 mSv, representing a significant reduction when compared to the radiation levels associated with standard-dose CT scans (Heltborg et al., 2024). Yan et al. (2021a) demonstrates the application of a CycleGAN model for denoising ultralow-dose CT images in evaluating PTB. The optimized CycleGAN model improved the peak signal-to-noise ratio by 2.0 dB and the structural similarity index by 0.21, providing satisfactory image quality with lower noise levels than hybrid and model-based iterative reconstruction techniques. The optimized CycleGAN technology might enable chest ULDCT to generate diagnostically acceptable images for TB evaluation.

### 5.5 Further expansion of PTB diagnosis

#### 5.5.1 Diagnosis of multidrug-resistant TB

Postprimary TB manifests in five distinct forms: infiltrative, focal, tuberculoma, miliary, and fibrocavernous. Multidrug-resistant PTB (MDR-PTB) often exhibits similar characteristics to those of drug-susceptible TB. DL techniques have demonstrated the potential to improve multidrug-resistant TB’s diagnostic precision and efficacy (MDR-TB). Gao and Qian (2018) using CT lung image data from a public dataset, a patch-based DL approach was proposed to classify multidrug-resistant TB and drug-sensitive TB. The CNN allied to the SVM classifier achieved an accuracy of 91.11% with the patch-based DL technique. This study overcame the challenge of a limited dataset of only 230 samples by using patches instead of full images, effectively expanding the dataset from hundreds to thousands. Duwairi and Melhem (2023) employed multi-channel models that incorporated image frames, mask frames, and gender/age data as inputs, utilizing transfer learning based on VGG19 and ResNet neural networks for feature extraction from CT scans. Their study’s best-performing model for MDR classification achieved an accuracy of 74.13% and an AUC of 64.2%. DeepTB is a DL system created using CNN-ResNet to learn transfer learning. It can quickly diagnose DR-TB and divide it into three main types: rifampicin-RTB, MDR-TB, and extensively drug-RTB. Utilizing complex network structures, DeepTB transforms input data into target predictions, achieving high performance for DR-TB diagnosis (AUC: 0.943). The model also attained an AUC of 0.880 for RR-TB, 0.928 for MDR-TB, and 0.918 for XDR-TB. Integrating class activation maps (CAMs) offers a visual explanation of the decision-making process, addressing the “black-box” issue of CNNs and boosting clinical trust in the system’s outputs (Liang et al., 2024).

#### 5.5.2 Diagnosing the infectiousness of PTB

The utilization of DL models in the analysis of CT images has demonstrated considerable potential in differentiating the infectivity of PTB patients. Gao et al. (2023) created a DL model called TBINet, which employs a 2D projection-based CNNs to assess the infectivity of PTB patients using CT images. The algorithm was trained on a dataset of 925 individuals from four sites, with infectivity classified according to several sputum samples conducted within a month. The TBiNet model exhibited enhanced performance, achieving an AUC of 0.753 on the external test set, surpassing current DL methodologies. Gradient-weighted class activation mapping (Grad-CAM) technology indicated that CT scans exhibiting increased consolidation, voids, upper lobe involvement, and larger lymph nodes were more frequently associated with patients suffering from highly infectious types of PTB.

#### 5.5.3 Diagnosing comorbidities of PTB

The diagnosis of comorbidities in patients with PTB is crucial for effective management and treatment. Several studies have highlighted the significance of identifying comorbidities such as diabetes mellitus and HIV infection in recently diagnosed PTB individuals (Sama et al., 2023). When diagnosing PTB, it is crucial to take into account a variety of factors, including age, sex, previous TB treatment history, and other comorbidities (Jiang et al., 2024). Additional research and guidelines are required to improve the diagnosis and management of comorbidities in PTB patients.

### 5.6 Integration with clinical decision support systems

While the initial validation results are impressive, further prospective validation studies are necessary within actual clinical settings. Once these commercial AI systems have been thoroughly tested, they could offer physicians globally convenient, efficient, and precise diagnostic tools, thereby aiding clinical decision-making in the foreseeable future. Integrating clinical decision support systems is a key challenge in applying DL to CT-based PTB diagnosis. This requires seamlessly integrating AI models with existing healthcare systems to ensure that diagnostic results are effectively communicated to physicians and influence clinical decision-making. Specifically, it requires addressing several challenges: First, integrating the output of AI models with data from existing systems like electronic medical records and PACS to ensure diagnostic results are associated with the patient’s other clinical information. This requires addressing issues around data formats and security. Second, ensuring AI-assisted diagnosis can seamlessly integrate into the physician’s clinical workflow without adding extra steps or disrupting the normal diagnostic process. This requires optimizing and redesigning existing workflows. Finally, physicians must understand the rationale and logic behind the AI model’s diagnoses to evaluate the results and perform secondary confirmations. This requires improving the interpretability of the AI model so that physicians can gain insights into its inner workings. Interpretability remains a critical challenge in the domain of CT-based pneumonia and PTB diagnosis, as DL models used for image analysis are often regarded as “black boxes” due to their high-dimensional and non-linear nature. Providing clear and actionable explanations can help physicians and DL models jointly improve diagnostic accuracy, reducing the risk of misdiagnosis of PTB. One effective approach involves using Grad-CAM to visualize the regions of the lungs that the model focuses on during the diagnosis of PTB, such as lesions or areas of consolidation (Jegatheeswaran et al., 2024). Additionally, Shapley additive explanations or local interpretable model-agnostic explanations can be applied to identify the most significant features contributing to the model’s predictions, such as pixel intensities or specific ROIs (Chung et al., 2024; Peng et al., 2024). Interactive tools like heatmaps can further enhance interpretability by allowing physicians to explore the model’s behavior and examine specific predictions in detail (Liu et al., 2020). It is also important to evaluate interpretability methods with physicians to ensure that the explanations are comprehensible and clinically relevant. Finally, ensuring compliance with regulatory standards and incorporating feedback from medical professionals will further support the safe and effective deployment of these systems in actual clinical settings.

## 6 Discussion

Data scarcity is a significant challenge in medical imaging, particularly for diseases like PTB, where annotated CT scans are limited. One potential solution is to employ a GAN framework to create synthetic CT images like real-world data features. Current research indicates that models trained on synthetic data can perform comparable to those trained on real data alone (Ali et al., 2024). Various geometric transformations, such as rotations, scaling, and flipping, help the model learn invariant features crucial for accurate diagnosis across different patient presentations. These transformations enhance the model’s ability to recognize disease patterns despite patient positioning and imaging technique variations (Mastouri et al., 2024). Transfer learning represents another promising strategy to address data scarcity (Wajgi et al., 2024). Researchers can initialize the network weights and fine-tune the model on the PTB CT data by leveraging pre-trained models on large-scale medical imaging datasets or even non-medical image datasets. For instance, models pre-trained in general lung disease detection tasks may have learned useful low-level and mid-level features such as lung structure identification and texture analysis. These pre-trained features can be transferred and further adapted to the specific task of TB diagnosis, thereby enhancing the model’s performance on limited datasets. To prevent overfitting and reduce model complexity, regularization techniques such as dropout and weight decay improve the model’s ability to generalize to new data by discouraging the model from relying too heavily on any training example. Future studies could focus on developing more advanced data augmentation techniques that mimic real-world variations in imaging data. Additionally, exploring hybrid transfer learning methods that combine multiple pre-trained models could optimize generalization for PTB diagnosis. Such approaches may lead to more robust and accurate diagnostic models, even in limited training data.

The application of DL in clinical settings has revolutionized healthcare, offering promising advancements in diagnostics. However, this technological leap also presents many ethical concerns that require careful consideration and resolution.

First, using DL in healthcare often involves processing sensitive patient data, raising concerns about privacy and security. To address this, robust encryption, anonymization, and secure data-sharing protocols are proposed to protect patient data (Mirzaei et al., 2024). Additionally, federated learning techniques are being explored to train models on decentralized data, which can help preserve privacy while allowing for practical model training (Mukund et al., 2024). Second, DL models can be affected by biases in the training data, which can cause doctors to make bad decisions. To fix this problem, researchers use data augmentation, data balance, and fairness-aware training to ensure that models accurately represent diverse groups of people and do not make differences worse. This method is essential for ensuring that DL systems are trustworthy in clinical settings. Third, the intricacy of DL models, frequently called “black boxes,” makes it challenging to understand and interpret how they make decisions fully. In clinical settings, the lack of transparency in the predictions made by algorithms can lead to skepticism among medical professionals regarding the model’s reliability (Rajpurkar et al., 2022). Scientists are creating explainable AI methods like saliency maps and attention processes to improve explainability (Cerekci et al., 2024). These offer insights into model predictions and support the development of transparency and confidence in clinical decision-making. Finally, the algorithms fail to complete accuracy, and the accountability for any detrimental outcomes resulting from erroneous predictions remains ambiguous. This engenders uncertainty among physicians and patients. The imprecise accuracy of AI systems presents significant responsibility concerns related to harm (Morin-Martel, 2023). This requires careful evaluation of DL’s diagnostic accuracy and its impact on clinical workflows, ensuring the technology is practical and ethically integrated into healthcare practices.

The accuracy of DL models in TB diagnosis is essential, as misdiagnoses can have severe consequences. Rigorous testing and validation of DL models against gold standards are required to guarantee accuracy. Consistently enhance the models to accommodate the evolving clinical landscape and emerging scientific findings. Innovative models must be evaluated in real-world medical settings and integrated smoothly into the standard operational procedures, particularly in nations with a high TB burden and limited access to sophisticated medical technology and specialized medical personnel to guide clinical practice effectively. Additionally, improving patient comprehension of the diagnostic procedure and clinician trust are benefits of developing explainable AI approaches for DL models used in TB diagnosis. These factors are critical for properly implementing these technologies in clinical settings.

Two frameworks were recommended to ensure the ethical application of DL in diagnosing PTB. The Principles of Biomedical Ethics serve as a foundational guide, emphasizing four central bioethical principles: autonomy, beneficence, non-maleficence, and justice (Mirzaei et al., 2024). These principles are crucial for evaluating the ethical implications of DL applications in the diagnosis of PTB, ensuring that they benefit patients without causing harm, respecting patient autonomy, and promoting equitable access to care. Furthermore, the Trustworthy AI Framework provides a comprehensive set of criteria for AI systems (Schwabe et al., 2024). It highlights the importance of human agency and oversight, diversity, non-discrimination, and fairness, underscoring the necessity for DL systems to be designed and deployed trustworthy to respect human rights.

## 7 Conclusion

Artificial intelligence-based techniques, such as DL and other traditional ML algorithms, when applied to PTB, offer an autonomous, convenient, and efficient approach to enhance diagnostic precision and speed, often surpassing the capabilities of radiologists. This study underscores the complexity involved in diagnosing PTB. It emphasizes the significant role of sophisticated DL and imaging diagnostic methods. The main goal of medical image processing is to use algorithms to get accurate and valuable information out of images with as little mistake as possible. The segmenting, classifying, and diagnosing PTB utilizing CT data generally comprises four essential stages: data acquisition and preprocessing, feature extraction, and classification. Furthermore, it is imperative to prioritize the interpretability of DL models when they are implemented in clinical decision-making processes. The research may be improved by examining the integration of multi-modal datasets and deploying real-time DL solutions in healthcare environments. Therefore, DL tools can be considered a promising diagnostic resource for PTB and various other life-threatening diseases.

## Data Availability

The original contributions presented in this study are included in this article/supplementary material, further inquiries can be directed to the corresponding authors.

## Appendix

**APPENDIX TABLE A1:** Evaluation of bias risk and applicability in selected studies.

Study	Risk of bias				Applicability concerns		
	**Patient selection**	**Index test**	**Reference standard**	**Flow and timing**	**Patient selection**	**Index test**	**Reference standard**
Zhang et al., 2024	Unclear risk	Low risk	Low risk	Unclear risk	Unclear risk	Low concern	Low concern
Haq et al., 2022	Unclear risk	Low risk	Low risk	Unclear risk	Unclear risk	Low concern	Low concern
Ma et al., 2020	Low risk	Low risk	Low risk	Unclear risk	Low risk	Low concern	Low concern
Huang et al., 2023	Low risk	Low risk	Low risk	Unclear risk	Low risk	Low concern	Low concern
Zhang et al., 2020	Low risk	Low risk	Low risk	Unclear risk	Low risk	Low concern	Low concern
Li X. K. et al., 2021	Unclear risk	Low risk	Low risk	Unclear risk	Unclear risk	Low concern	Low concern
Lu et al., 2022	Unclear risk	Low risk	Low risk	Unclear risk	Unclear risk	Low concern	Low concern
